# Psychological safety, job satisfaction, and the intention to leave among German early-career physicians

**DOI:** 10.1093/intqhc/mzaf002

**Published:** 2025-01-17

**Authors:** Nicola Etti, Matthias Weigl, Nikoloz Gambashidze

**Affiliations:** Institute for Patient Safety, University Hospital Bonn, 53127 Bonn, Nordrhein-Westfalen, Germany; Städtisches Klinikum Solingen GmbH, Gotenstr.1, Solingen 42653, Germany; Institute for Patient Safety, University Hospital Bonn, 53127 Bonn, Nordrhein-Westfalen, Germany; Institute for Patient Safety, University Hospital Bonn, 53127 Bonn, Nordrhein-Westfalen, Germany

**Keywords:** psychological safety, job satisfaction, intention to leave, physicians

## Abstract

**Background:**

Healthcare systems worldwide experience shortages of healthcare professionals. Retention of physicians is becoming an increasing problem. The psychological safety among physicians affects not only performance but also their emotional well-being and job satisfaction. This study aims to evaluate early career physicians’ perception of psychological safety and its influence on job satisfaction and intention to leave.

**Methods:**

In a cross-sectional study, early career physicians, currently in fellowship programs in Germany were invited to fill in an electronic survey. The instrument consisted of demographic variables and sections from validated and well-established questionnaires. Psychological safety was evaluated on three levels—in relation to the team leader, team as a whole, and peers. Also, job satisfaction was assessed with standardized measures, and participants were asked if they were considering leaving their current employer. Participants were recruited via a nationwide learning platform—an online educational portal for medical students and early career physicians. Data analyses included descriptive, correlation analysis, and regression analyses to determine univariate and multivariate associations with job satisfaction and intention to leave.

**Results:**

The study sample consisted of 432 early career physicians. Most were fulltime employed (85.6%), female (78.2%), and in first 3 years of their postgraduate education (77.5%). A total of 47.2% indicated intention to leave their current employment. On a Likert-10 agreement scale, with high scores indicating greater psychological safety, the mean scores for leader-related, team-related, and peer-related psychological safety were 6.01 [95% confidence interval = 5.81–6.21), 7.30 (7.11–7.49), and 7.95 (7.78–8.12), respectively. In correlation analysis, all dimensions of psychological safety showed significant associations with job satisfaction and the intention to leave. In the multiple regression analyses, female gender (B = −0.10; *P* = .04) and age group (B = −0.08; *P* < .01) were associated with lower job satisfaction. High leader and team-related psychological safety were significantly associated with higher job satisfaction (B = 0.18, *P* < .01; B = 0.10, *P* < .01), and negatively related to intention to leave (OR = 0.53, *P* < 0.01; OR = 0.77, *P* < .01).

**Conclusion:**

This survey enhances our understanding of the nuances of psychological safety among early career physicians. In Germany, they reported low-to-medium levels of psychological safety related to the leader and low job satisfaction. Almost every second participant indicated intention to leave the organization. Leader-related psychological safety had highest effect on job satisfaction and intention to leave. Our findings corroborate the eminent role of leadership, workplace, and safety culture for job satisfaction and retention of early career physicians, what consequently affects quality and safety of healthcare.

## Introduction

Physician turnover and retention is an increasing challenge for healthcare systems worldwide [1]. In Germany, studies have indicated that a substantial percentage of physicians (up to 29%), are contemplating leaving their current positions [2, 3]. A recent study found that up to 28% of young physicians in Germany indicated high intention to leave the profession [4]. High turnover rates are associated with intensive costs, diminished productivity and poorer patient outcomes [3]. These results underscore the urgent need to address the physician turnover to enhance the stability and effectiveness of healthcare systems.

Understanding the factors that contribute to physician turnover is critical for developing effective retention strategies. Previous research has indicated various organizational factors associated with physician turnover or the intention to leave. High-quality teamwork, work-related support from supervisors and peers, and alignment of personal and organizational values have been linked with decreased physician turnover [2, 5]. Conversely, burnout and lack of professional fulfilment increase the likelihood of physicians leaving [5]. A study of young physicians and nurses showed that the quality of care and job satisfaction have significant effect on their intention to leave the profession [4].

Psychological safety is ‘a shared belief that the team is safe for interpersonal risk taking’ [6]. It is a critical facilitator of safe work environments, allowing team members to communicate openly, voice concerns, and discuss problems without fear of retribution [7]. Team members develop a shared sense of interpersonal safety [6], which affects job satisfaction, work engagement, organizational commitment, and burnout [7, 8]. According to a concept analysis, the individual, interpersonal and organizational factors support psychological safety in healthcare settings [8]. Downstream effects of psychological safety include outcomes like quality of care and patient safety, inter-professional collaboration and innovation, job satisfaction, work engagement, and organizational commitment [9]. These outcomes are essential for maintaining a stable and effective healthcare workforce.

Direct effect of the different dimensions of psychological safety on the intention to leave among physicians is hardly studied. Given the key potential of psychological safety to enhance job satisfaction and reduce burnout, exploring its direct relationships with physicians’ turnover intentions could provide valuable insights for healthcare management.

## Objectives

This study aims to examine perceptions of different dimensions of psychological safety and respective associations with job satisfaction and the intention to leave among early-career physicians. More specifically, we aimed to investigate:

Q1. What is the level of perceived psychological safety, job satisfaction, and the intention to leave among early-career physicians in Germany?

Q2. How do different dimensions of psychological safety affect job satisfaction and the intention to leave, respectively?

## Methods

### Setting and participants

In this cross-sectional study, early career physicians were invited to participate in an online survey. The sample was collected in collaboration with Medi-Learn, a German online educational platform for medical students and early career physicians [10]. The membership is free and not limited to a certain subspecialty. All registered physicians of Medi-Learn database in current German fellowship training were included. Fellowship training in German healthcare system usually encompasses specialty training for 5–6 years after graduation. Participation was anonymous and detailed study information, data protection policies, and consent forms were provided before the online survey. The study has been approved by the Ethics Committee of the Medical Faculty of the University of Bonn (387/22-EP). To support transparent reporting of findings, we used STROBE guidelines for cross-sectional surveys [11].

### Data collection

On 16 December 2022 all physicians in fellowship registered in the Medi-Learn database (*N* > 25 000) were invited via standardized email. The invitation included information about the survey and the data security, as well as the link to the anonymous online survey. On 10 March 2023, active users (those having showed any activity in the online educational programs between 1 January 2022 and 1 March 2023; *N* = 2417) received an additional email reminder. Data were collected using online survey platform (Unipark, Tivian XI GmbH). The survey was anonymous and data were processed by the research team.

### Measures

The study instrument consisted of selected dimensions from validated and well-established questionnaires. The questionnaire covered also other constructs, which are not included in the current analysis. Before data collection, the authors of the instruments were informed about the use of their scales and the translation if needed. Instruments not available in German were translated into German by the authors and back-translated by an independent translation agency to ensure meaning was expressed as in the original. All items were administrated in German language (English translations in text below are provided for the publication transparency). Beyond study constructs, we collected information on demographic variables, including age, sex, clinical area, year of fellowship, tenure at the current employer, full- or part-time employment, and organization ownership.

#### Psychological safety

Psychological safety was measured using the 19-item survey instrument developed by O’Donovan *et al*. [33]. This scale measures three, distinct dimensions of psychological safety in relation to (I) the team leader, (i the team as a whole, and (iii) other team members (peers). Examples of the questions include ‘If I had a question or was unsure of something in relation to my role at work, I could ask my team leader’, and ‘It is easy to ask other members of this team for help’. The items were measured using a Likert-10 agreement scale (1-Strongly disagree; 10-Strongly agree), with higher scores indicating higher psychological safety.

#### Job satisfaction

Job satisfaction was measured using the German version of Copenhagen Psychosocial Questionnaire (COPSOQ) [12]. The seven items of the instrument measure satisfaction regarding different aspects of work (e.g. ‘Considering your work situation as a whole, how satisfied are you with the people you work with?’). We used a four-point Likert scale (1-very unsatisfied; 4-very satisfied), with higher scores indicating higher job satisfaction.

#### The intention to leave

The intention to leave the organization was captured using a single item, asking participants if they intended to leave or already left their current employer due to dissatisfaction with organizational culture (‘I am so dissatisfied with the working atmosphere at my job that I am thinking about resigning or have already resigned’). Participants could disagree (score 0) or agree (score 1) to the statement.

### Data analysis

Before the main analysis, we evaluated internal consistency of scales using Cronbach’s alpha, interpreting values >0.7 as ‘acceptable’ and >0.8 as good [13]. The dimension scores were formed by aggregating means of corresponding items. To answer the first research question (Q1), we calculated mean scores and 95% confidence intervals of the three dimensions of psychological safety and job satisfaction. For the dichotomous intention to leave, we calculated the frequency of positive responses. To evaluate the differences between psychological safety related to the leader, team and peers, we used paired samples *t*-tests. Correlations between variables were evaluated using Spearman’s correlations. To answer the second study question (Q2), linear and logistic multiple regression were used to evaluate the associations with the three dimensions of psychological safety (independent variables) with job satisfaction and intention to leave (dependent variables). Demographic variables were included in the regression analyses as covariates. Before the regression analyses, we evaluated presence of multicollinearity by measuring the Variance Inflammation Factor (VIF) for each independent variable, expecting it to be below the required threshold of 10 [13]. Data were analysed using IBM SPSS Statistics 27.0.

## Results

### The sample

In the first wave, all 25 129 fellow physicians registered in the Medi-Learn system were invited to participate in the survey. In the second wave, 2417 active users (participating in one or more online trainings between 1 January 2022 and 1 March 2023) received a reminder. Finally, 432 early career physicians completed the entire questionnaire (1.7% of all registered users, 17.8% of active users).

The sample for analysis consisted of 432 physicians. Among those, 338 were female (78.2%). Majority (54.6%) were between 25 and 30 years of age. Most participants were in their first three years of postgraduate education, with 23.8%, 28.9%, and 24.8%, respectively. Most participants (85.6%) worked fulltime. Employment tenure was mainly between 1 and 3 years. The majority of participants were employed at general hospitals (61.3%) or university hospitals (24.5%). Hospital ownership status was mostly public (50.0%), followed by private (17.4%), and nonprofit (14.4%). The demographic data are presented in Table 1.

**Table 1.** Demographic characteristics of the sample.

	*N*	%
Total sample	432	100.0
Sex		
Male	88	20.4
Female	338	78.2
Other/no reply	6	1.4
**Age group, years; Mean, (SD)**	31.03 (4.42)	
25–30	236	54.6
31–35	133	30.8
36–40	50	11.6
≥40	13	3.0
**Fellowship year**		
1st year	103	23.8
2nd year	125	28.9
3rd year	107	24.8
4th year	62	14.4
5th year	29	6.7
6th year	6	1.4
**Current employment tenure**		
Less than 6 months	67	15.5
6–11 months	86	19.9
1–3 years	210	48.6
More than 3 years	64	14.8
Missing/no reply	5	1.2
**Employed**		
Full-time	370	85.6
Part time	62	14.4
**Clinical area**		
Internal Medicine	121	28.0
Anaesthesiology	58	13.4
Surgery	53	12.3
Paediatrics	45	10.4
General Medicine	30	6.9
Other	125	28.9%
**Employer**		
University Hospital	106	24.5
General Hospital	265	61.3
Other	52	12.0
Missing/no reply	9	2.1
**Organization ownership**		
Public	216	50.0
Nonprofit	62	14.4
Private	75	17.4
Missing/no reply	79	18.3

### Descriptive statistics of study variables

As stated in Table 2, mean scores for the three dimensions of psychological safety related to the leader, team, and peers were 6.01, 7.30, and 7.95, respectively. According to the paired sample *t*-test, all differences between psychological safety dimensions were statistically significant (*P* < 0.05). Among study participants, 47.22% indicated intention to leave their current employment. Mean score for the job satisfaction was 2.58.

**Table 2.** Descriptive results and reliability of job satisfaction and psychological safety scales.

	Scale	Mean	(95% CI)	Cronbach’s alpha
Job satisfaction	1–4	2.58	(2.53–2.64)	0.873
Psychological safety (PS)				
Leader-related PS	1–10	6.01	(5.81–6.21)	0.945
Team-related PS	1–10	7.30	(7.11–7.49)	0.863
Peer-related PS	1–10	7.95	(7.78–8.12)	0.951

Note: *N* = 432, CI—Confidence interval.

### Bivariate analyses of study variables

Correlations between variables are presented in Table 3. Participants’ gender and fulltime employment were not associated with any of the other variables. Participants’ age group, fellowship year and employment tenure were all positively correlated, and had mostly significant negative correlations with dimensions of psychological safety and job satisfaction. Fellowship year and employment tenure also had significant positive correlation with intention to leave. Intention to leave negatively correlated with the job satisfaction as well as with all three dimensions of psychological safety, respectively. Associations between job satisfaction and the dimensions of psychological safety ranged between 0.473 and 0.728.

**Table 3.** Spearman’s correlations among study variables.

	b	*N*	1	2	3	4	5	6	7	8	9
1	Female gender ^a^	426									
2	Age group	432	−0.076								
3	Fellowship year	432	0.036	0.277**							
4	Tenure	427	0.069	0.147**	0.606**						
5	Fulltime employment ^b^	432	−0.060	−0.123*	−0.042	-,087					
	PS										
6	Leader-related PS	432	0.094	−0.109*	−0.116*	−0.124*	0.026				
7	Team-related PS	432	0.009	−0.189**	−0.105*	−0.135**	0.055	0.559**			
8	Peer-related PS	432	0.020	−0.186**	−0.058	−0.045	0.052	0.579**	0.707**		
	Outcome variables										
9	Intention to leave	432	0.001	0.073	0.150**	0.170**	−0.010	−0.551**	−0.403**	−0.317**	
10	Job satisfaction	432	−0.008	−0.190**	−0.112*	−0.149**	0.024	0.728**	0.538**	0.473**	−0.684**

Note:

* *P* < .05;

** *P* < .01.

^a^Reference group ‘male’; ^b^reference group ‘part time’. 1—Female gender; 2—Age group; 3—Fellowship year; 4—Tenure; 5—Fulltime employment; 6—Leader related Psychological Safety; 7—Team related Psychological Safety; 8—Peer related Psychological Safety; 9—Intention to leave; 10—Job Satisfaction.

### Multiple regression analyses

To evaluate associations of the three dimensions of psychological safety with job satisfaction and intention to leave, we used multiple linear regression model and multiple logistic regression models, respectively. In both multivariate models, the three dimensions of the psychological safety were included as independent variables. Demographic variables were female gender, age group, fellowship year, tenure at the organisation and fulltime employment and included as covariates (cf., Table 4). The VIFs for the independent variables ranged from 1.03 to 2.30, well below the threshold of 10, indicating that the independent variables did not exhibit problematic levels of collinearity.

**Table 4.** Multiple regressions of psychological safety dimensions with job satisfaction, and intention to leave.

	Job satisfaction (Multiple linear regression)				Intention to leave (Multiple logistic regression)			
	B	95% CI		*P*-value	OR	95% CI		*P*-value
**Constant**	1.36	1.10	1.62	.00	37.16	-	-	.00
Female gender	**−0.10**	**−0.21**	**0.00**	**.04**	1.42	0.78	2.58	.25
Age group	**−0.08**	**−0.13**	**−0.03**	**.00**	0.91	0.66	1.27	.59
Fellowship year	0.02	−0.02	0.06	.29	1.10	0.87	1.41	.42
Tenure	−0.04	−0.09	0.01	.12	1.27	0.92	1.76	.15
Fulltime employment	−0.01	−0.12	0.10	.83	1.20	0.61	2.37	.60
**PS**								
Leader-related PS	**0.18**	**0.15**	**0.20**	**.00**	**0.53**	**0.45**	**0.63**	**.00**
Team-related PS	**0.05**	**0.02**	**0.08**	**.00**	**0.77**	**0.64**	**0.92**	**.00**
Peer-related PS	0.01	−0.03	0.04	.64	1.10	0.90	1.35	.35
**Adjusted *R*^2^**	**0.576**				**0.432^a^**			

Note: *N* = 421; PS—psychological safety; CI—confidence interval; a—Nagelkerke R Square; Significant effects (*P* < .05) are in bold.

In the multiple linear regression analysis, lower job satisfaction was associated with female gender (B = −0.10, 95% CI −0.21 to 0.00, *P* = .04) and higher age group (B = −0.08, 95% CI −0.13 to 0.03, *P* < .01). Among the dimensions of psychological safety, positive effects on job satisfaction were leader-related (B = 0.18, 95% CI 0.15 to 0.20, *P* < .01) and team-related psychological safety (B = 0.05, 95% CI 0.02 to 0.08, *P* < .01). The relationship with peer-related psychological safety was not significant.

The multiple logistic retrogression analysis showed significant negative effect of leader-related [odds ratio (OR) = 0.53, 95% CI 0.45 to 0.63, *P* < .01] and team-related psychological safety (OR = 0.77, 95% CI 0.64 to 0.92, *P* < .01), indicating lower intention to leave with higher scores on psychological safety. Peer-related psychological safety as well as demographic variables did not have significant effects on the intention to leave.

## Discussion

### Statement of principal findings

This study provides novel insights into early-career physicians’ perceptions of psychological safety and examines its effect on job satisfaction and intention to leave. Previous studies have reported associations of psychological safety with various outcomes related to physician wellbeing, job satisfaction, and performance. These outcomes in turn have been associated with physician turnover. We aimed to close the gap, by capturing distinct aspects of psychological safety in the clinical workplace and evaluating direct effects the safety dimensions on intention to leave among early-career physicians.

### Interpretation within the context of wider literature

#### Psychological safety, job satisfaction, and intention to leave among junior physicians

Overall, participants reported medium to high levels of psychological safety. Interestingly, psychological safety related to the leader was lowest, followed by the team related, and, with highest scores, peer-related psychological safety. Mean score for job satisfaction was also moderate, indicating considerable room for improvement. Unexpectedly, increases in age, tenure, and year of fellowship were all related to lower perceptions of psychological safety. All these are usually followed by increased expertise, associated with higher work autonomy as well as less hierarchical levels, which have been described previously as antecedents of psychological safety [14, 15]. Post-hoc, one possible explanation might be the social experiences within clinical teams. If surveyed junior physicians experience limited support when asking questions, admitting mistakes or expressing new ideas, their perceptions of psychological safety tend to decrease [16]. Similar results could be found in team projects, where psychological safety at team level declined over time [17]. It has been argued, that team members at early stages feel safe to ask questions, whereas at later stages questions, ideas and critique might feel unsafe due to possible retarding effects on projects’ progress. We assume, that with accumulating time, young physicians may also feel pressure to demonstrate more knowledge and clinical experience, to ask fewer questions, work more independently and make less mistakes. Consequently, with time, asking for help or admitting mistakes might feel less safe in teams. In such an environment, counterintuitively, more experienced team members may need more support to facilitate psychological safety.

Within a new team, members do not have personal impressions, and fresh doctors may be carrying perceptions of psychological safety over from their previous medical studies. Through every day and direct social interactions specific experiences shape team climate and perceptions of psychological safety [17]. Consequently, an alternative explanation for lower psychological safety scores associated with time and experience in our results may be nonfavourable social interactions along the medical practice, experiences, which decrease perceptions of psychological safety.

Another important finding of our study was that psychological safety related to peers scored highest, while psychological safety related to team leader scored lowest. This might indicate the need to take a closer look on leadership behaviours in healthcare institutions—in Germany as well as elsewhere. Leader’s role in workplace culture has been widely discussed in studies over recent decades [18]. Positive leader behaviours, inclusiveness, behavioural integrity and change orientation have shown positive effects on psychological safety of team members [19]. Also low power distance showed similar effects [14], while psychological safety-mediated effects between power distance and perceived team effectiveness [20]. Leader’s behaviour, their reaction on speaking up, and their alignment of norms and values, shape workplace culture and perceptions of psychological safety [16]. In learning environments, leaders promote acquisition of theoretical knowledge and practical skills, but also influence norms, values, and behaviours, and, ultimately, shaping workplace culture [21]. Disruptive behaviour in contrast, either on leader or peer level, can lead to dysfunctional cultures [22]. In clinical work environments, leader’s behaviour, their reaction on speaking up can be vital for perceptions of psychological safety [16]. Our findings underscore the key role of positive leader behaviour for learning, performance and psychological outcomes like job satisfaction, burnout, and intention to leave. Leadership trainings raising awareness and mentorship programmes for emergent leaders could contribute to favourable workplace behaviours and may promote successful interaction across hierarchical boundaries.

#### The effect of psychological safety dimensions on job satisfaction and intention to leave

Our study provides novel insights into the concerted or joint effects of distinct dimensions of psychological safety in the clinical workplace and their interplay with physician outcomes. In the adjusted, multivariate analyses, only psychological safety related to the leader and to the team revealed highly significant associations with job satisfaction and intention to leave. Yet, psychological safety related to peers showed no significant effect. In previous publications supportive leadership, organizational support and alignment of personal and organizational norms and values, as well as support on peer level were identified as influence factors on supportive social work contexts [5] with favourable effects on job satisfaction and the intention to leave [2, 15]. Leaders seem to play an eminent role in shaping work environments [19] as they form employee’s expectations on social behaviour within a group [23]. A spectrum of leadership behaviours has been described as determinants of psychological safety, including inclusiveness, accessibility, acknowledgment of fallibility and integrity [19, 24, 25].

In our study, female gender was associated with lower job satisfaction. This resonates well with a previous study among physicians analysing various domains of job satisfaction [26], reporting that female physicians were less satisfied, which might be caused by work-life imbalance, or limited control over work planning. With almost half of participants in our study expressing explicitly intention to leave and moderate levels of job satisfaction, German early-career physicians appear to be in need of supportive work system interventions, promoting physician work conditions and well-being on the job [27]. Addressing leadership behaviour might be one pertinent way to create safe work cultures and avoid increased costs, loss of expertise and reduced patient safety due to turnover [7]. With physician turnover becoming prevalent issue worldwide, further local and international studies could provide deeper insights into the effects of psychological safety on job satisfaction and intention to leave among physicians, as well as the influences of broader cultural- and healthcare-related factors.

#### Implications for policy, practice, and research

Our results indicate alarmingly high levels of intention to leave among early-career German physicians (i.e. almost half of study participants). Improvement in work environment and job satisfaction show positive effect on employee turnover [7, 28], and fostering psychological safety is pertinent to promote retention on the job. High scores of peer-related psychological safety stood in contrast to lower values of psychological safety related to the leader. This might indicate the need to focus on leadership issues in the hospital and improve leadership behaviours of superiors. Leaders have shown to play a salient role shaping climates within work groups [9, 19]. Various favourable leadership behaviours, including inclusiveness [29] authenticity and behavioural integrity [30] and a transformational leadership style have been described in literature and could be integrated in educational interventions for supervisors.

In addition to improvements in leadership styles, opportunities to enhance the work environment and promote professional retention can include fostering awareness of psychological safety within clinical teams, and detecting and preventing disruptive behaviours. Moreover, interventions to increase job satisfaction and physician retention could target physicians’ working conditions, including excessive job demands, opportunities for self-determination, work-life balance, economic factors, and professional development opportunities [3, 7, 26].

### Strength and limitations

This study provides a novel contribution by exploring the associations between psychological safety and intention to leave among early-career physicians in Germany. The use of validated and well-established instruments ensures the reliability and comparability of the findings. Additionally, the multidimensional approach to psychological safety (leader, team, and peers) allows a deeper understanding of its specific impacts.

The results of this cross-sectional study need to be interpreted in light of some limitations. First, our sample consisted of the registered and, most likely, active users of an online educational platform. Although the platform has a high reputation and its services are widely used among junior physicians throughout Germany, it is difficult to infer on representability of the sample. Our sample had a high proportion of female respondents, which is in line with current trends in gender distribution according to the German registry of physicians [31]. Statistics show rising proportions of females among medical students and early career physicians over the last decades [32].

Second, as participation was voluntary and without remuneration, self-selection bias may have occurred, i.e. as those with interest in the subject of our research may have been more inclined to participate. To mitigate this bias and to facilitate participation, we collected the data anonymously, provided broad time window for participation, and sent out a reminder to participate. Future studies may further facilitate recruitment (e.g. with reasonable incentives), or employ other methods for data collection.

Third, our study design draws upon physicians’ self-reports and does not allow for detailed insights into psychological safety and leadership styles within clinical teams, as would an observational or in-depth qualitative study [33]. Moreover, the inclusion of objective data on actual physician turnover could provide additional insight into outcomes, rather than relying solely on self-reports. We acknowledge that our nationwide evaluation, may neglect differences in psychological safety attributed to teams from different healthcare sectors, specialties, or facilities. Finally, our analysis is limited by the cross-sectional study design.

## Conclusion

Based on our findings, almost half of the surveyed early-career physicians indicated intention to leave the organization due to the poor organizational culture. Physicians’ level of psychological safety related to peers was significantly higher compared to psychological safety related to the leader and to the team. With accumulated time during specialty training, early career physicians seem to perceive lower psychological safety and job satisfaction, and higher intention to leave the organization. Our multivariate analyses revealed that psychological safety related to leader had highest effect on job satisfaction and the intention to leave. Our findings contribute to a better and nuanced understanding into the different dimension of psychological safety among junior physicians. It may further inform design of future interventions and unfolding opportunities to improve psychological safety and facilitate retention of healthcare professionals.

## Data Availability

Data will not be made available in the public domain. Access to the data can be requested through a reasonable request to the corresponding author or formal collaboration, ensuring compliance with national and regional data security standards.
